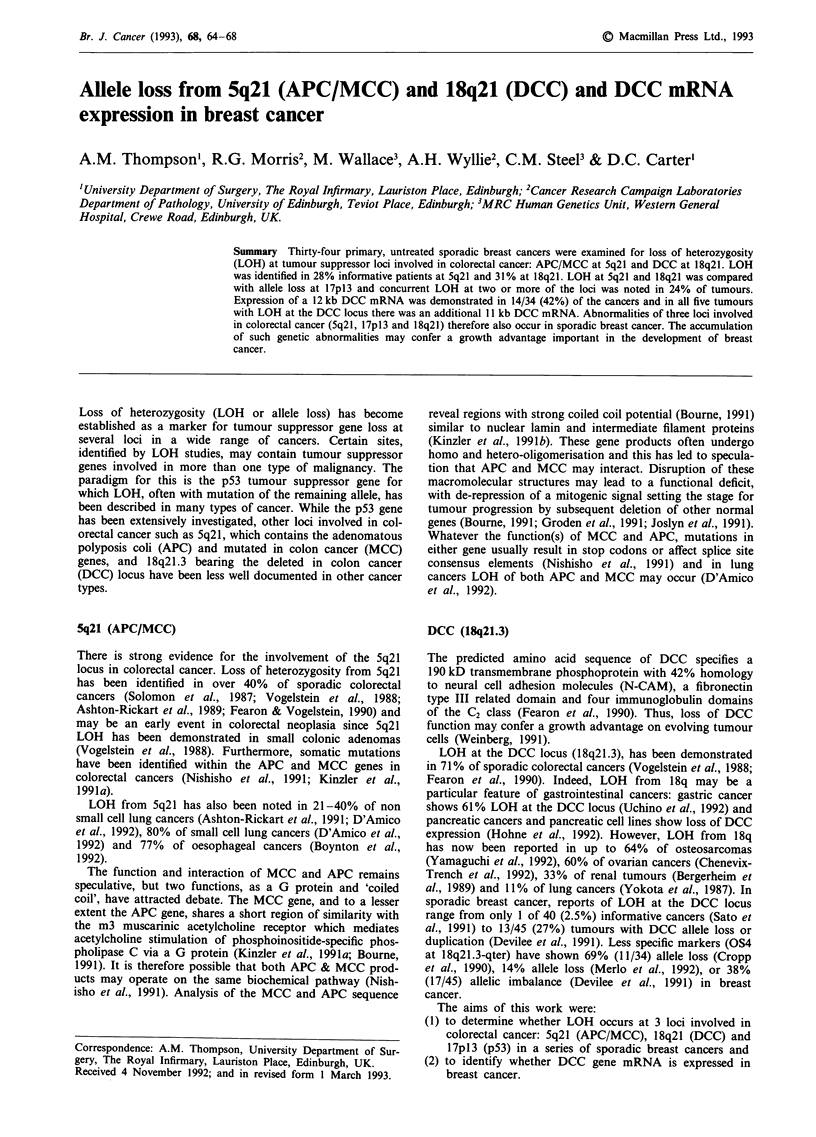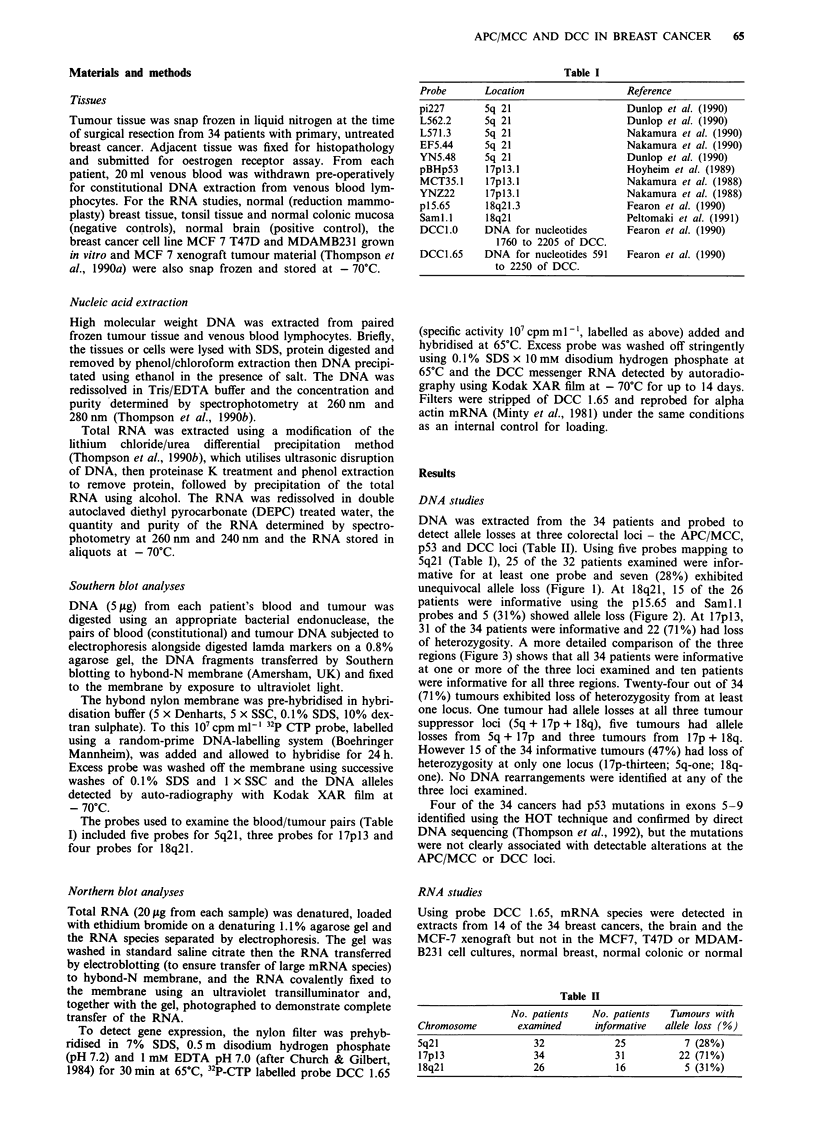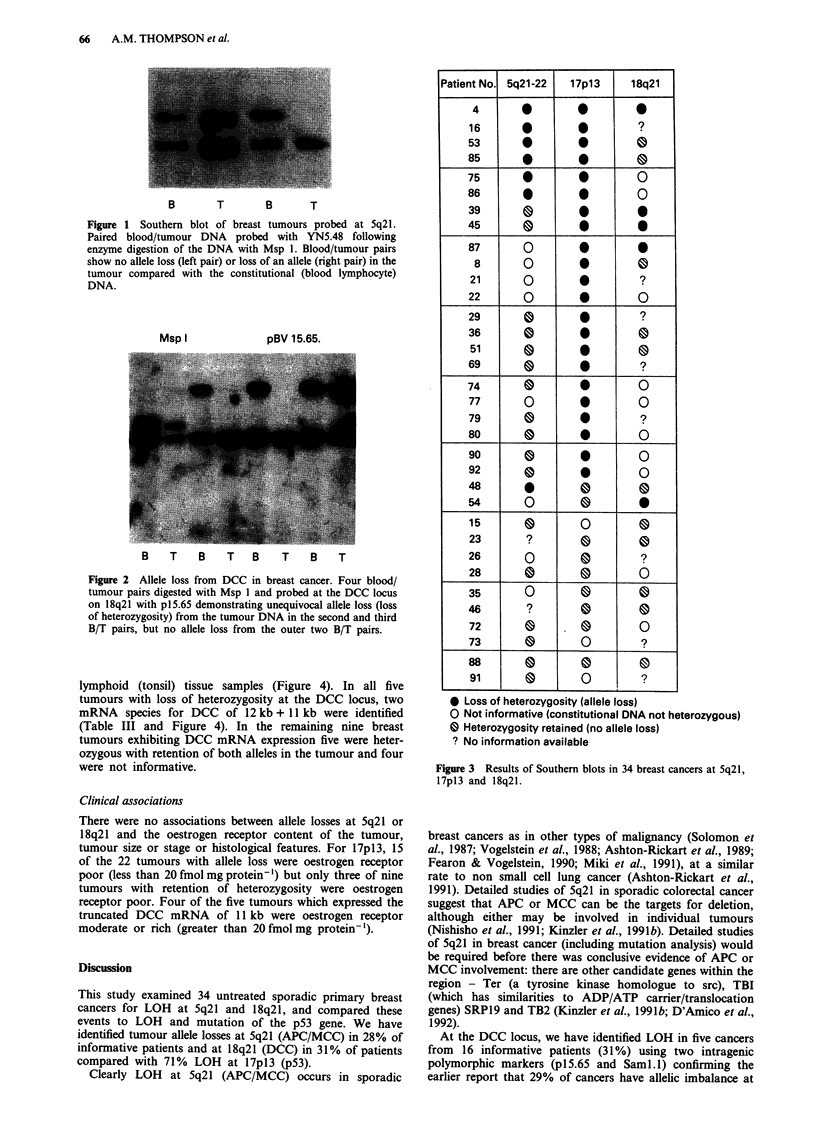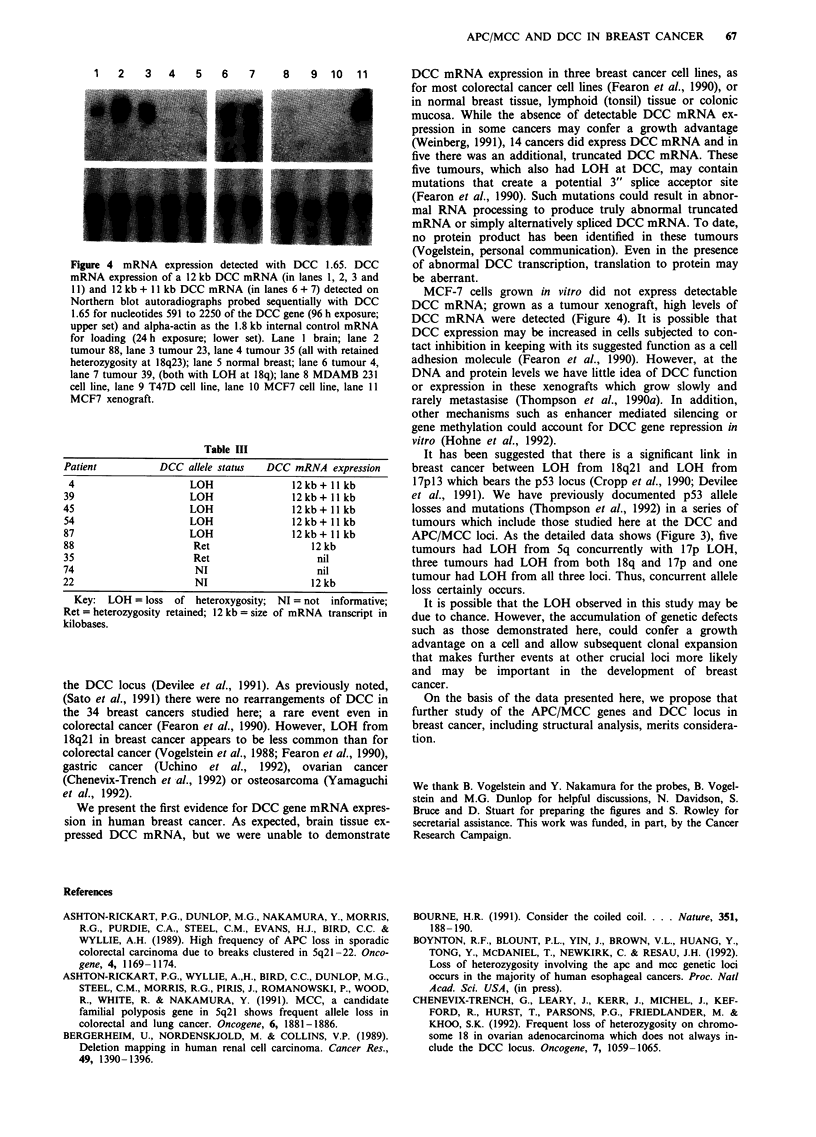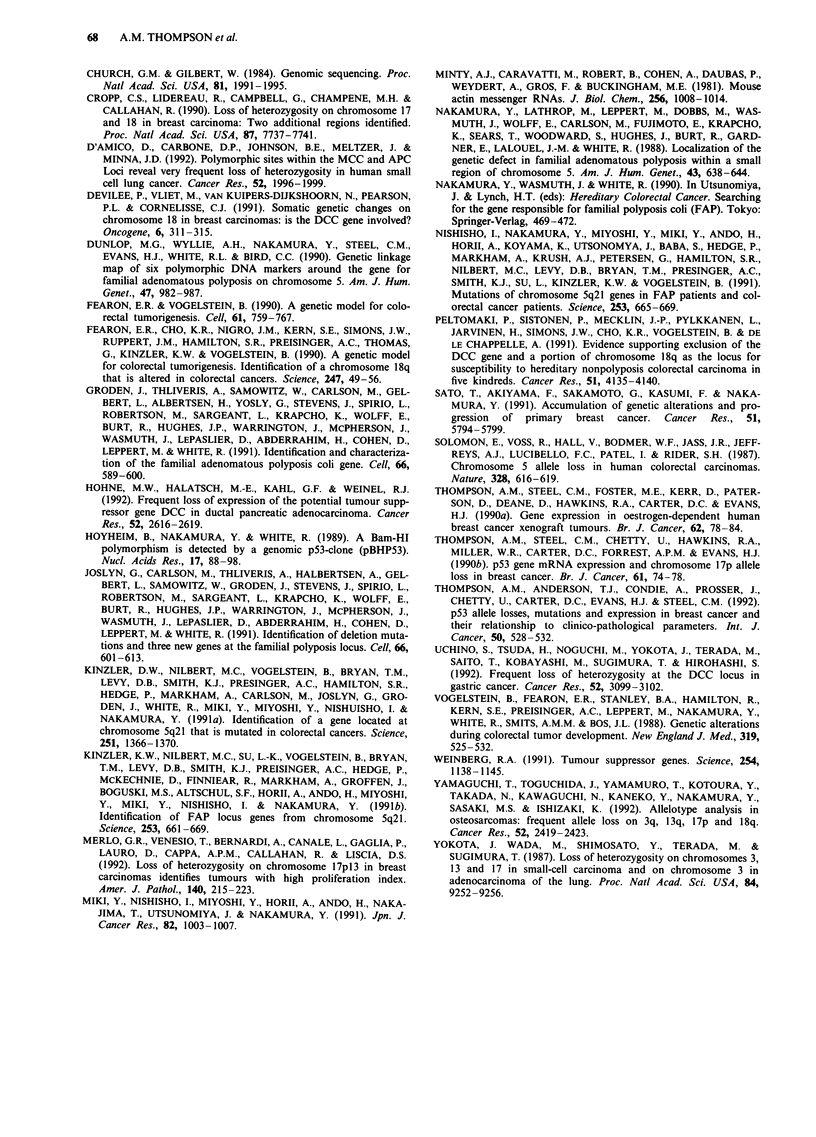# Allele loss from 5q21 (APC/MCC) and 18q21 (DCC) and DCC mRNA expression in breast cancer.

**DOI:** 10.1038/bjc.1993.287

**Published:** 1993-07

**Authors:** A. M. Thompson, R. G. Morris, M. Wallace, A. H. Wyllie, C. M. Steel, D. C. Carter

**Affiliations:** University Department of Surgery, Royal Infirmary, Edinburgh, UK.

## Abstract

**Images:**


Br. J. Cancer (1993), 68, 64-68 © Macmillan Press Ltd., 1993

### Allele loss from 5q21 (APC/MCC) and 18q21 (DCC) and DCC mRNA expression in breast cancer

A.M. Thompson[1], R.G. Morris[2], M. Wallace[3], A.H. Wyllie[2], C.M. Steel[3] & D.C. Carter[1]

[1]*University Department of Surgery, The Royal Infirmary, Lauriston Place, Edinburgh;* [2]*Cancer Research Campaign Laboratories Department of Pathology, University of Edinburgh, Teviot Place, Edinburgh;* [3]*MRC Human Genetics Unit, Western General Hospital, Crewe Road, Edinburgh, UK.*

**Summary** Thirty-four primary, untreated sporadic breast cancers were examined for loss of heterozygosity (LOH) at tumour suppressor loci involved in colorectal cancer: APC/MCC at 5q21 and DCC at 18q21. LOH was identified in 28% informative patients at 5q21 and 31% at 18q21. LOH at 5q21 and 18q21 was compared with allele loss at 17p13 and concurrent LOH at two or more of the loci was noted in 24% of tumours. Expression of a 12 kb DCC mRNA was demonstrated in 14/34 (42%) of the cancers and in all five tumours with LOH at the DCC locus there was an additional 11 kb DCC mRNA. Abnormalities of three loci involved in colorectal cancer (5q21, 17p13 and 18q21) therefore also occur in sporadic breast cancer. The accumulation of such genetic abnormalities may confer a growth advantage important in the development of breast cancer.

Loss of heterozygosity (LOH or allele loss) has become established as a marker for tumour suppressor gene loss at several loci in a wide range of cancers. Certain sites, identified by LOH studies, may contain tumour suppressor genes involved in more than one type of malignancy. The paradigm for this is the p53 tumour suppressor gene for which LOH, often with mutation of the remaining allele, has been described in many types of cancer. While the p53 gene has been extensively investigated, other loci involved in colorectal cancer such as 5q21, which contains the adenomatous polyposis coli (APC) and mutated in colon cancer (MCC) genes, and 18q21.3 bearing the deleted in colon cancer (DCC) locus have been less well documented in other cancer types.

### 5q21 (APC/MCC)

There is strong evidence for the involvement of the 5q21 locus in colorectal cancer. Loss of heterozygosity from 5q21 has been identified in over 40% of sporadic colorectal cancers (Solomon *et al.*, 1987; Vogelstein *et al.*, 1988; Ashton-Rickart *et al.*, 1989; Fearon & Vogelstein, 1990) and may be an early event in colorectal neoplasia since 5q21 LOH has been demonstrated in small colonic adenomas (Vogelstein *et al.*, 1988). Furthermore, somatic mutations have been identified within the APC and MCC genes in colorectal cancers (Nishisho *et al.*, 1991; Kinzler *et al.*, 1991*a*).

LOH from 5q21 has also been noted in 21-40% of non small cell lung cancers (Ashton-Rickart *et al.*, 1991; D'Amico *et al.*, 1992), 80% of small cell lung cancers (D'Amico *et al.*, 1992) and 77% of oesophageal cancers (Boynton *et al.*, 1992).

The function and interaction of MCC and APC remains speculative, but two functions, as a G protein and 'coiled coil', have attracted debate. The MCC gene, and to a lesser extent the APC gene, shares a short region of similarity with the m3 muscarinic acetylcholine receptor which mediates acetylcholine stimulation of phosphoinositide-specific phospholipase C via a G protein (Kinzler *et al.*, 1991*a*; Bourne, 1991). It is therefore possible that both APC & MCC products may operate on the same biochemical pathway (Nishisho *et al.*, 1991). Analysis of the MCC and APC sequence reveal regions with strong coiled coil potential (Bourne, 1991) similar to nuclear lamin and intermediate filament proteins (Kinzler *et al.*, 1991*b*). These gene products often undergo homo and hetero-oligomerisation and this has led to speculation that APC and MCC may interact. Disruption of these macromolecular structures may lead to a functional deficit, with de-repression of a mitogenic signal setting the stage for tumour progression by subsequent deletion of other normal genes (Bourne, 1991; Groden *et al.*, 1991; Joslyn *et al.*, 1991). Whatever the function(s) of MCC and APC, mutations in either gene usually result in stop codons or affect splice site consensus elements (Nishisho *et al.*, 1991) and in lung cancers LOH of both APC and MCC may occur (D'Amico *et al.*, 1992).

### DCC (18q21.3)

The predicted amino acid sequence of DCC specifies a 190 kD transmembrane phosphoprotein with 42% homology to neural cell adhesion molecules (N-CAM), a fibronectin type III related domain and four immunoglobulin domains of the $C_2$ class (Fearon *et al.*, 1990). Thus, loss of DCC function may confer a growth advantage on evolving tumour cells (Weinberg, 1991).

LOH at the DCC locus (18q21.3), has been demonstrated in 71% of sporadic colorectal cancers (Vogelstein *et al.*, 1988; Fearon *et al.*, 1990). Indeed, LOH from 18q may be a particular feature of gastrointestinal cancers: gastric cancer shows 61% LOH at the DCC locus (Uchino *et al.*, 1992) and pancreatic cancers and pancreatic cell lines show loss of DCC expression (Hohne *et al.*, 1992). However, LOH from 18q has now been reported in up to 64% of osteosarcomas (Yamaguchi *et al.*, 1992), 60% of ovarian cancers (Chenevix-Trench *et al.*, 1992), 33% of renal tumours (Bergerheim *et al.*, 1989) and 11% of lung cancers (Yokota *et al.*, 1987). In sporadic breast cancer, reports of LOH at the DCC locus range from only 1 of 40 (2.5%) informative cancers (Sato *et al.*, 1991) to 13/45 (27%) tumours with DCC allele loss or duplication (Devilee *et al.*, 1991). Less specific markers (OS4 at 18q21.3-qter) have shown 69% (11/34) allele loss (Cropp *et al.*, 1990), 14% allele loss (Merlo *et al.*, 1992), or 38% (17/45) allelic imbalance (Devilee *et al.*, 1991) in breast cancer.

The aims of this work were:
(1) to determine whether LOH occurs at 3 loci involved in colorectal cancer: 5q21 (APC/MCC), 18q21 (DCC) and 17p13 (p53) in a series of sporadic breast cancers and
(2) to identify whether DCC gene mRNA is expressed in breast cancer.

Correspondence: A.M. Thompson, University Department of Surgery, The Royal Infirmary, Lauriston Place, Edinburgh, UK.
Received 4 November 1992; and in revised form 1 March 1993.

### Materials and methods

### *Tissues*

Tumour tissue was snap frozen in liquid nitrogen at the time of surgical resection from 34 patients with primary, untreated breast cancer. Adjacent tissue was fixed for histopathology and submitted for oestrogen receptor assay. From each patient, 20 ml venous blood was withdrawn pre-operatively for constitutional DNA extraction from venous blood lymphocytes. For the RNA studies, normal (reduction mammoplasty) breast tissue, tonsil tissue and normal colonic mucosa (negative controls), normal brain (positive control), the breast cancer cell line MCF 7 T47D and MDAMB231 grown *in vitro* and MCF 7 xenograft tumour material (Thompson *et al.*, 1990*a*) were also snap frozen and stored at − 70°C.

### *Nucleic acid extraction*

High molecular weight DNA was extracted from paired frozen tumour tissue and venous blood lymphocytes. Briefly, the tissues or cells were lysed with SDS, protein digested and removed by phenol/chloroform extraction then DNA precipitated using ethanol in the presence of salt. The DNA was redissolved in Tris/EDTA buffer and the concentration and purity determined by spectrophotometry at 260 nm and 280 nm (Thompson *et al.*, 1990*b*).

Total RNA was extracted using a modification of the lithium chloride/urea differential precipitation method (Thompson *et al.*, 1990*b*), which utilises ultrasonic disruption of DNA, then proteinase K treatment and phenol extraction to remove protein, followed by precipitation of the total RNA using alcohol. The RNA was redissolved in double autoclaved diethyl pyrocarbonate (DEPC) treated water, the quantity and purity of the RNA determined by spectrophotometry at 260 nm and 240 nm and the RNA stored in aliquots at − 70°C.

### *Southern blot analyses*

DNA (5 μg) from each patient's blood and tumour was digested using an appropriate bacterial endonuclease, the pairs of blood (constitutional) and tumour DNA subjected to electrophoresis alongside digested lamda markers on a 0.8% agarose gel, the DNA fragments transferred by Southern blotting to hybond-N membrane (Amersham, UK) and fixed to the membrane by exposure to ultraviolet light.

The hybond nylon membrane was pre-hybridised in hybridisation buffer (5 × Denharts, 5 × SSC, 0.1% SDS, 10% dextran sulphate). To this $10^7$ cpm $ml^{-1}$ $^{32}P$ CTP probe, labelled using a random-prime DNA-labelling system (Boehringer Mannheim), was added and allowed to hybridise for 24 h. Excess probe was washed off the membrane using successive washes of 0.1% SDS and 1 × SSC and the DNA alleles detected by auto-radiography with Kodak XAR film at − 70°C.

The probes used to examine the blood/tumour pairs (Table I) included five probes for 5q21, three probes for 17p13 and four probes for 18q21.

### *Northern blot analyses*

Total RNA (20 μg from each sample) was denatured, loaded with ethidium bromide on a denaturing 1.1% agarose gel and the RNA species separated by electrophoresis. The gel was washed in standard saline citrate then the RNA transferred by electroblotting (to ensure transfer of large mRNA species) to hybond-N membrane, and the RNA covalently fixed to the membrane using an ultraviolet transilluminator and, together with the gel, photographed to demonstrate complete transfer of the RNA.

To detect gene expression, the nylon filter was prehybridised in 7% SDS, 0.5 m disodium hydrogen phosphate (pH 7.2) and 1 mM EDTA pH 7.0 (after Church & Gilbert, 1984) for 30 min at 65°C, $^{32}P$-CTP labelled probe DCC 1.65 (specific activity $10^7$ cpm $ml^{-1}$, labelled as above) added and hybridised at 65°C. Excess probe was washed off stringently using 0.1% SDS × 10 mM disodium hydrogen phosphate at 65°C and the DCC messenger RNA detected by autoradiography using Kodak XAR film at − 70°C for up to 14 days. Filters were stripped of DCC 1.65 and reprobed for alpha actin mRNA (Minty *et al.*, 1981) under the same conditions as an internal control for loading.

**Table I**

| *Probe* | *Location* | *Reference* |
|---|---|---|
| pi227 | 5q 21 | Dunlop *et al.* (1990) |
| L562.2 | 5q 21 | Dunlop *et al.* (1990) |
| L571.3 | 5q 21 | Nakamura *et al.* (1990) |
| EF5.44 | 5q 21 | Nakamura *et al.* (1990) |
| YN5.48 | 5q 21 | Dunlop *et al.* (1990) |
| pBHp53 | 17p13.1 | Hoyheim *et al.* (1989) |
| MCT35.1 | 17p13.1 | Nakamura *et al.* (1988) |
| YNZ22 | 17p13.1 | Nakamura *et al.* (1988) |
| p15.65 | 18q21.3 | Fearon *et al.* (1990) |
| Sam1.1 | 18q21 | Peltomaki *et al.* (1991) |
| DCC1.0 | DNA for nucleotides 1760 to 2205 of DCC. | Fearon *et al.* (1990) |
| DCC1.65 | DNA for nucleotides 591 to 2250 of DCC. | Fearon *et al.* (1990) |

### Results

### *DNA studies*

DNA was extracted from the 34 patients and probed to detect allele losses at three colorectal loci – the APC/MCC, p53 and DCC loci (Table II). Using five probes mapping to 5q21 (Table I), 25 of the 32 patients examined were informative for at least one probe and seven (28%) exhibited unequivocal allele loss (Figure 1). At 18q21, 15 of the 26 patients were informative using the p15.65 and Sam1.1 probes and 5 (31%) showed allele loss (Figure 2). At 17p13, 31 of the 34 patients were informative and 22 (71%) had loss of heterozygosity. A more detailed comparison of the three regions (Figure 3) shows that all 34 patients were informative at one or more of the three loci examined and ten patients were informative for all three regions. Twenty-four out of 34 (71%) tumours exhibited loss of heterozygosity from at least one locus. One tumour had allele losses at all three tumour suppressor loci (5q + 17p + 18q), five tumours had allele losses from 5q + 17p and three tumours from 17p + 18q. However 15 of the 34 informative tumours (47%) had loss of heterozygosity at only one locus (17p-thirteen; 5q-one; 18q-one). No DNA rearrangements were identified at any of the three loci examined.

Four of the 34 cancers had p53 mutations in exons 5–9 identified using the HOT technique and confirmed by direct DNA sequencing (Thompson *et al.*, 1992), but the mutations were not clearly associated with detectable alterations at the APC/MCC or DCC loci.

### *RNA studies*

Using probe DCC 1.65, mRNA species were detected in extracts from 14 of the 34 breast cancers, the brain and the MCF-7 xenograft but not in the MCF7, T47D or MDAM-B231 cell cultures, normal breast, normal colonic or normal

**Table II**

| *Chromosome* | *No. patients examined* | *No. patients informative* | *Tumours with allele loss (%)* |
|---|---|---|---|
| 5q21 | 32 | 25 | 7 (28%) |
| 17p13 | 34 | 31 | 22 (71%) |
| 18q21 | 26 | 16 | 5 (31%) |

**Figure 1** Southern blot of breast tumours probed at 5q21. Paired blood/tumour DNA probed with YN5.48 following enzyme digestion of the DNA with Msp 1. Blood/tumour pairs show no allele loss (left pair) or loss of an allele (right pair) in the tumour compared with the constitutional (blood lymphocyte) DNA.

**Figure 2** Allele loss from DCC in breast cancer. Four blood/tumour pairs digested with Msp 1 and probed at the DCC locus on 18q21 with p15.65 demonstrating unequivocal allele loss (loss of heterozygosity) from the tumour DNA in the second and third B/T pairs, but no allele loss from the outer two B/T pairs.

| Patient No. | 5q21-22 | 17p13 | 18q21 |
|---|---|---|---|
| 4 | ● | ● | ● |
| 16 | ● | ● | ? |
| 53 | ● | ● | ⊘ |
| 85 | ● | ● | ⊘ |
| 75 | ● | ● | O |
| 86 | ● | ● | O |
| 39 | ⊘ | ● | ● |
| 45 | ⊘ | ● | ● |
| 87 | O | ● | ● |
| 8 | O | ● | ⊘ |
| 21 | O | ● | ? |
| 22 | O | ● | O |
| 29 | ⊘ | ● | ? |
| 36 | ⊘ | ● | ⊘ |
| 51 | ⊘ | ● | ⊘ |
| 69 | ⊘ | ● | ? |
| 74 | ⊘ | ● | O |
| 77 | O | ● | O |
| 79 | ⊘ | ● | ? |
| 80 | ⊘ | ● | O |
| 90 | ⊘ | ● | O |
| 92 | ⊘ | ● | O |
| 48 | ● | ⊘ | ⊘ |
| 54 | O | ⊘ | ● |
| 15 | ⊘ | O | ⊘ |
| 23 | ? | ⊘ | ⊘ |
| 26 | O | ⊘ | ? |
| 28 | ⊘ | ⊘ | O |
| 35 | O | ⊘ | ⊘ |
| 46 | ? | ⊘ | ⊘ |
| 72 | ⊘ | ⊘ | O |
| 73 | ⊘ | O | ? |
| 88 | ⊘ | ⊘ | ⊘ |
| 91 | ⊘ | O | ? |

● Loss of heterozygosity (allele loss)
O Not informative (constitutional DNA not heterozygous)
⊘ Heterozygosity retained (no allele loss)
? No information available

**Figure 3** Results of Southern blots in 34 breast cancers at 5q21, 17p13 and 18q21.

lymphoid (tonsil) tissue samples (Figure 4). In all five tumours with loss of heterozygosity at the DCC locus, two mRNA species for DCC of 12 kb + 11 kb were identified (Table III and Figure 4). In the remaining nine breast tumours exhibiting DCC mRNA expression five were heterozygous with retention of both alleles in the tumour and four were not informative.

*Clinical associations*

There were no associations between allele losses at 5q21 or 18q21 and the oestrogen receptor content of the tumour, tumour size or stage or histological features. For 17p13, 15 of the 22 tumours with allele loss were oestrogen receptor poor (less than 20 fmol mg protein$^{-1}$) but only three of nine tumours with retention of heterozygosity were oestrogen receptor poor. Four of the five tumours which expressed the truncated DCC mRNA of 11 kb were oestrogen receptor moderate or rich (greater than 20 fmol mg protein$^{-1}$).

### Discussion

This study examined 34 untreated sporadic primary breast cancers for LOH at 5q21 and 18q21, and compared these events to LOH and mutation of the p53 gene. We have identified tumour allele losses at 5q21 (APC/MCC) in 28% of informative patients and at 18q21 (DCC) in 31% of patients compared with 71% LOH at 17p13 (p53).

Clearly LOH at 5q21 (APC/MCC) occurs in sporadic breast cancers as in other types of malignancy (Solomon et al., 1987; Vogelstein et al., 1988; Ashton-Rickart et al., 1989; Fearon & Vogelstein, 1990; Miki et al., 1991), at a similar rate to non small cell lung cancer (Ashton-Rickart et al., 1991). Detailed studies of 5q21 in sporadic colorectal cancer suggest that APC or MCC can be the targets for deletion, although either may be involved in individual tumours (Nishisho et al., 1991; Kinzler et al., 1991b). Detailed studies of 5q21 in breast cancer (including mutation analysis) would be required before there was conclusive evidence of APC or MCC involvement: there are other candidate genes within the region – Ter (a tyrosine kinase homologue to src), TBI (which has similarities to ADP/ATP carrier/translocation genes) SRP19 and TB2 (Kinzler et al., 1991b; D'Amico et al., 1992).

At the DCC locus, we have identified LOH in five cancers from 16 informative patients (31%) using two intragenic polymorphic markers (p15.65 and Sam1.1) confirming the earlier report that 29% of cancers have allelic imbalance at

**Figure 4** mRNA expression detected with DCC 1.65. DCC mRNA expression of a 12 kb DCC mRNA (in lanes 1, 2, 3 and 11) and 12 kb + 11 kb DCC mRNA (in lanes 6 + 7) detected on Northern blot autoradiographs probed sequentially with DCC 1.65 for nucleotides 591 to 2250 of the DCC gene (96 h exposure; upper set) and alpha-actin as the 1.8 kb internal control mRNA for loading (24 h exposure; lower set). Lane 1 brain; lane 2 tumour 88, lane 3 tumour 23, lane 4 tumour 35 (all with retained heterozygosity at 18q23); lane 5 normal breast; lane 6 tumour 4, lane 7 tumour 39, (both with LOH at 18q); lane 8 MDAMB 231 cell line, lane 9 T47D cell line, lane 10 MCF7 cell line, lane 11 MCF7 xenograft.

**Table III**

| *Patient* | *DCC allele status* | *DCC mRNA expression* |
|---|---|---|
| 4 | LOH | 12 kb + 11 kb |
| 39 | LOH | 12 kb + 11 kb |
| 45 | LOH | 12 kb + 11 kb |
| 54 | LOH | 12 kb + 11 kb |
| 87 | LOH | 12 kb + 11 kb |
| 88 | Ret | 12 kb |
| 35 | Ret | nil |
| 74 | NI | nil |
| 22 | NI | 12 kb |

Key: LOH = loss of heteroxygosity; NI = not informative; Ret = heterozygosity retained; 12 kb = size of mRNA transcript in kilobases.

the DCC locus (Devilee *et al.*, 1991). As previously noted, (Sato *et al.*, 1991) there were no rearrangements of DCC in the 34 breast cancers studied here; a rare event even in colorectal cancer (Fearon *et al.*, 1990). However, LOH from 18q21 in breast cancer appears to be less common than for colorectal cancer (Vogelstein *et al.*, 1988; Fearon *et al.*, 1990), gastric cancer (Uchino *et al.*, 1992), ovarian cancer (Chenevix-Trench *et al.*, 1992) or osteosarcoma (Yamaguchi *et al.*, 1992).

We present the first evidence for DCC gene mRNA expression in human breast cancer. As expected, brain tissue expressed DCC mRNA, but we were unable to demonstrate DCC mRNA expression in three breast cancer cell lines, as for most colorectal cancer cell lines (Fearon *et al.*, 1990), or in normal breast tissue, lymphoid (tonsil) tissue or colonic mucosa. While the absence of detectable DCC mRNA expression in some cancers may confer a growth advantage (Weinberg, 1991), 14 cancers did express DCC mRNA and in five there was an additional, truncated DCC mRNA. These five tumours, which also had LOH at DCC, may contain mutations that create a potential 3" splice acceptor site (Fearon *et al.*, 1990). Such mutations could result in abnormal RNA processing to produce truly abnormal truncated mRNA or simply alternatively spliced DCC mRNA. To date, no protein product has been identified in these tumours (Vogelstein, personal communication). Even in the presence of abnormal DCC transcription, translation to protein may be aberrant.

MCF-7 cells grown *in vitro* did not express detectable DCC mRNA; grown as a tumour xenograft, high levels of DCC mRNA were detected (Figure 4). It is possible that DCC expression may be increased in cells subjected to contact inhibition in keeping with its suggested function as a cell adhesion molecule (Fearon *et al.*, 1990). However, at the DNA and protein levels we have little idea of DCC function or expression in these xenografts which grow slowly and rarely metastasise (Thompson *et al.*, 1990*a*). In addition, other mechanisms such as enhancer mediated silencing or gene methylation could account for DCC gene repression *in vitro* (Hohne *et al.*, 1992).

It has been suggested that there is a significant link in breast cancer between LOH from 18q21 and LOH from 17p13 which bears the p53 locus (Cropp *et al.*, 1990; Devilee *et al.*, 1991). We have previously documented p53 allele losses and mutations (Thompson *et al.*, 1992) in a series of tumours which include those studied here at the DCC and APC/MCC loci. As the detailed data shows (Figure 3), five tumours had LOH from 5q concurrently with 17p LOH, three tumours had LOH from both 18q and 17p and one tumour had LOH from all three loci. Thus, concurrent allele loss certainly occurs.

It is possible that the LOH observed in this study may be due to chance. However, the accumulation of genetic defects such as those demonstrated here, could confer a growth advantage on a cell and allow subsequent clonal expansion that makes further events at other crucial loci more likely and may be important in the development of breast cancer.

On the basis of the data presented here, we propose that further study of the APC/MCC genes and DCC locus in breast cancer, including structural analysis, merits consideration.

We thank B. Vogelstein and Y. Nakamura for the probes, B. Vogelstein and M.G. Dunlop for helpful discussions, N. Davidson, S. Bruce and D. Stuart for preparing the figures and S. Rowley for secretarial assistance. This work was funded, in part, by the Cancer Research Campaign.

### References

ASHTON-RICKART, P.G., DUNLOP, M.G., NAKAMURA, Y., MORRIS, R.G., PURDIE, C.A., STEEL, C.M., EVANS, H.J., BIRD, C.C. & WYLLIE, A.H. (1989). High frequency of APC loss in sporadic colorectal carcinoma due to breaks clustered in 5q21-22. *Oncogene*, **4**, 1169-1174.

ASHTON-RICKART, P.G., WYLLIE, A.,H., BIRD, C.C., DUNLOP, M.G., STEEL, C.M., MORRIS, R.G., PIRIS, J., ROMANOWSKI, P., WOOD, R., WHITE, R. & NAKAMURA, Y. (1991). MCC, a candidate familial polyposis gene in 5q21 shows frequent allele loss in colorectal and lung cancer. *Oncogene*, **6**, 1881-1886.

BERGERHEIM, U., NORDENSKJOLD, M. & COLLINS, V.P. (1989). Deletion mapping in human renal cell carcinoma. *Cancer Res.*, **49**, 1390-1396.

BOURNE, H.R. (1991). Consider the coiled coil. . . . *Nature*, **351**, 188-190.

BOYNTON, R.F., BLOUNT, P.L., YIN, J., BROWN, V.L., HUANG, Y., TONG, Y., McDANIEL, T., NEWKIRK, C. & RESAU, J.H. (1992). Loss of heterozygosity involving the apc and mcc genetic loci occurs in the majority of human esophageal cancers. *Proc. Natl Acad. Sci. USA*, (in press).

CHENEVIX-TRENCH, G., LEARY, J., KERR, J., MICHEL, J., KEFFORD, R., HURST, T., PARSONS, P.G., FRIEDLANDER, M. & KHOO, S.K. (1992). Frequent loss of heterozygosity on chromosome 18 in ovarian adenocarcinoma which does not always include the DCC locus. *Oncogene*, **7**, 1059-1065.

CHURCH, G.M. & GILBERT, W. (1984). Genomic sequencing. *Proc. Natl Acad. Sci. USA*, **81**, 1991–1995.

CROPP, C.S., LIDEREAU, R., CAMPBELL, G., CHAMPENE, M.H. & CALLAHAN, R. (1990). Loss of heterozygosity on chromosome 17 and 18 in breast carcinoma: Two additional regions identified. *Proc. Natl Acad. Sci. USA*, **87**, 7737–7741.

D'AMICO, D., CARBONE, D.P., JOHNSON, B.E., MELTZER, J. & MINNA, J.D. (1992). Polymorphic sites within the MCC and APC Loci reveal very frequent loss of heterozygosity in human small cell lung cancer. *Cancer Res.*, **52**, 1996–1999.

DEVILEE, P., VLIET, M., VAN KUIPERS-DIJKSHOORN, N., PEARSON, P.L. & CORNELISSE, C.J. (1991). Somatic genetic changes on chromosome 18 in breast carcinomas: is the DCC gene involved? *Oncogene*, **6**, 311–315.

DUNLOP, M.G., WYLLIE, A.H., NAKAMURA, Y., STEEL, C.M., EVANS, H.J., WHITE, R.L. & BIRD, C.C. (1990). Genetic linkage map of six polymorphic DNA markers around the gene for familial adenomatous polyposis on chromosome 5. *Am. J. Hum. Genet.*, **47**, 982–987.

FEARON, E.R. & VOGELSTEIN, B. (1990). A genetic model for colorectal tumorigenesis. *Cell*, **61**, 759–767.

FEARON, E.R., CHO, K.R., NIGRO, J.M., KERN, S.E., SIMONS, J.W., RUPPERT, J.M., HAMILTON, S.R., PREISINGER, A.C., THOMAS, G., KINZLER, K.W. & VOGELSTEIN, B. (1990). A genetic model for colorectal tumorigenesis. Identification of a chromosome 18q that is altered in colorectal cancers. *Science*, **247**, 49–56.

GRODEN, J., THLIVERIS, A., SAMOWITZ, W., CARLSON, M., GELBERT, L., ALBERTSEN, H., YOSLY, G., STEVENS, J., SPIRIO, L., ROBERTSON, M., SARGEANT, L., KRAPCHO, K., WOLFF, E., BURT, R., HUGHES, J.P., WARRINGTON, J., MCPHERSON, J., WASMUTH, J., LEPASLIER, D., ABDERRAHIM, H., COHEN, D., LEPPERT, M. & WHITE, R. (1991). Identification and characterization of the familial adenomatous polyposis coli gene. *Cell*, **66**, 589–600.

HOHNE, M.W., HALATSCH, M.-E., KAHL, G.F. & WEINEL, R.J. (1992). Frequent loss of expression of the potential tumour suppressor gene DCC in ductal pancreatic adenocarcinoma. *Cancer Res.*, **52**, 2616–2619.

HOYHEIM, B., NAKAMURA, Y. & WHITE, R. (1989). A Bam-HI polymorphism is detected by a genomic p53-clone (pBHP53). *Nucl. Acids Res.*, **17**, 88–98.

JOSLYN, G., CARLSON, M., THLIVERIS, A., HALBERTSEN, A., GELBERT, L., SAMOWITZ, W., GRODEN, J., STEVENS, J., SPIRIO, L., ROBERTSON, M., SARGEANT, L., KRAPCHO, K., WOLFF, E., BURT, R., HUGHES, J.P., WARRINGTON, J., MCPHERSON, J., WASMUTH, J., LEPASLIER, D., ABDERRAHIM, H., COHEN, D., LEPPERT, M. & WHITE, R. (1991). Identification of deletion mutations and three new genes at the familial polyposis locus. *Cell*, **66**, 601–613.

KINZLER, D.W., NILBERT, M.C., VOGELSTEIN, B., BRYAN, T.M., LEVY, D.B., SMITH, K.J., PRESINGER, A.C., HAMILTON, S.R., HEDGE, P., MARKHAM, A., CARLSON, M., JOSLYN, G., GRODEN, J., WHITE, R., MIKI, Y., MIYOSHI, Y., NISHUISHO, I. & NAKAMURA, Y. (1991*a*). Identification of a gene located at chromosome 5q21 that is mutated in colorectal cancers. *Science*, **251**, 1366–1370.

KINZLER, K.W., NILBERT, M.C., SU, L.-K., VOGELSTEIN, B., BRYAN, T.M., LEVY, D.B., SMITH, K.J., PREISINGER, A.C., HEDGE, P., MCKECHNIE, D., FINNIEAR, R., MARKHAM, A., GROFFEN, J., BOGUSKI, M.S., ALTSCHUL, S.F., HORII, A., ANDO, H., MIYOSHI, Y., MIKI, Y., NISHISHO, I. & NAKAMURA, Y. (1991*b*). Identification of FAP locus genes from chromosome 5q21. *Science*, **253**, 661–669.

MERLO, G.R., VENESIO, T., BERNARDI, A., CANALE, L., GAGLIA, P., LAURO, D., CAPPA, A.P.M., CALLAHAN, R. & LISCIA, D.S. (1992). Loss of heterozygosity on chromosome 17p13 in breast carcinomas identifies tumours with high proliferation index. *Amer. J. Pathol.*, **140**, 215–223.

MIKI, Y., NISHISHO, I., MIYOSHI, Y., HORII, A., ANDO, H., NAKAJIMA, T., UTSUNOMIYA, J. & NAKAMURA, Y. (1991). *Jpn. J. Cancer Res.*, **82**, 1003–1007.

MINTY, A.J., CARAVATTI, M., ROBERT, B., COHEN, A., DAUBAS, P., WEYDERT, A., GROS, F. & BUCKINGHAM, M.E. (1981). Mouse actin messenger RNAs. *J. Biol. Chem.*, **256**, 1008–1014.

NAKAMURA, Y., LATHROP, M., LEPPERT, M., DOBBS, M., WASMUTH, J., WOLFF, E., CARLSON, M., FUJIMOTO, E., KRAPCHO, K., SEARS, T., WOODWARD, S., HUGHES, J., BURT, R., GARDNER, E., LALOUEL, J.-M. & WHITE, R. (1988). Localization of the genetic defect in familial adenomatous polyposis within a small region of chromosome 5. *Am. J. Hum. Genet.*, **43**, 638–644.

NAKAMURA, Y., WASMUTH, J. & WHITE, R. (1990). In Utsunomiya, J. & Lynch, H.T. (eds): *Hereditary Colorectal Cancer*. Searching for the gene responsible for familial polyposis coli (FAP). Tokyo: Springer-Verlag, 469–472.

NISHISHO, I., NAKAMURA, Y., MIYOSHI, Y., MIKI, Y., ANDO, H., HORII, A., KOYAMA, K., UTSONOMYA, J., BABA, S., HEDGE, P., MARKHAM, A., KRUSH, A.J., PETERSEN, G., HAMILTON, S.R., NILBERT, M.C., LEVY, D.B., BRYAN, T.M., PRESINGER, A.C., SMITH, K.J., SU, L., KINZLER, K.W. & VOGELSTEIN, B. (1991). Mutations of chromosome 5q21 genes in FAP patients and colorectal cancer patients. *Science*, **253**, 665–669.

PELTOMAKI, P., SISTONEN, P., MECKLIN, J.-P., PYLKKANEN, L., JARVINEN, H., SIMONS, J.W., CHO, K.R., VOGELSTEIN, B. & DE LE CHAPPELLE, A. (1991). Evidence supporting exclusion of the DCC gene and a portion of chromosome 18q as the locus for susceptibility to hereditary nonpolyposis colorectal carcinoma in five kindreds. *Cancer Res.*, **51**, 4135–4140.

SATO, T., AKIYAMA, F., SAKAMOTO, G., KASUMI, F. & NAKAMURA, Y. (1991). Accumulation of genetic alterations and progression of primary breast cancer. *Cancer Res.*, **51**, 5794–5799.

SOLOMON, E., VOSS, R., HALL, V., BODMER, W.F., JASS, J.R., JEFFREYS, A.J., LUCIBELLO, F.C., PATEL, I. & RIDER, S.H. (1987). Chromosome 5 allele loss in human colorectal carcinomas. *Nature*, **328**, 616–619.

THOMPSON, A.M., STEEL, C.M., FOSTER, M.E., KERR, D., PATERSON, D., DEANE, D., HAWKINS, R.A., CARTER, D.C. & EVANS, H.J. (1990*a*). Gene expression in oestrogen-dependent human breast cancer xenograft tumours. *Br. J. Cancer*, **62**, 78–84.

THOMPSON, A.M., STEEL, C.M., CHETTY, U., HAWKINS, R.A., MILLER, W.R., CARTER, D.C., FORREST, A.P.M. & EVANS, H.J. (1990*b*). p53 gene mRNA expression and chromosome 17p allele loss in breast cancer. *Br. J. Cancer*, **61**, 74–78.

THOMPSON, A.M., ANDERSON, T.J., CONDIE, A., PROSSER, J., CHETTY, U., CARTER, D.C., EVANS, H.J. & STEEL, C.M. (1992). p53 allele losses, mutations and expression in breast cancer and their relationship to clinico-pathological parameters. *Int. J. Cancer*, **50**, 528–532.

UCHINO, S., TSUDA, H., NOGUCHI, M., YOKOTA, J., TERADA, M., SAITO, T., KOBAYASHI, M., SUGIMURA, T. & HIROHASHI, S. (1992). Frequent loss of heterozygosity at the DCC locus in gastric cancer. *Cancer Res.*, **52**, 3099–3102.

VOGELSTEIN, B., FEARON, E.R., STANLEY, B.A., HAMILTON, R., KERN, S.E., PREISINGER, A.C., LEPPERT, M., NAKAMURA, Y., WHITE, R., SMITS, A.M.M. & BOS, J.L. (1988). Genetic alterations during colorectal tumor development. *New England J. Med.*, **319**, 525–532.

WEINBERG, R.A. (1991). Tumour suppressor genes. *Science*, **254**, 1138–1145.

YAMAGUCHI, T., TOGUCHIDA, J., YAMAMURO, T., KOTOURA, Y., TAKADA, N., KAWAGUCHI, N., KANEKO, Y., NAKAMURA, Y., SASAKI, M.S. & ISHIZAKI, K. (1992). Allelotype analysis in osteosarcomas: frequent allele loss on 3q, 13q, 17p and 18q. *Cancer Res.*, **52**, 2419–2423.

YOKOTA, J. WADA, M., SHIMOSATO, Y., TERADA, M. & SUGIMURA, T. (1987). Loss of heterozygosity on chromosomes 3, 13 and 17 in small-cell carcinoma and on chromosome 3 in adenocarcinoma of the lung. *Proc. Natl Acad. Sci. USA*, **84**, 9252–9256.